# The Therapeutical Value of Nitro-Glycerine

**Published:** 1880-11

**Authors:** 


THE THERAPEUTICAL VALUE OF NITRO-GLYCERINE.

A. W. Mayo Robson, F. R. C. S., of Leeds, writes as follows to the *British Medical Journal*, April 10, 1880:

During the last twelve months I have tried this remedy in migraine, asthma, angina pectoris and epilepsy. In migraine, one or two drops of a one-per-cent. solution produces, within a few minutes, a diminution of tension in the previously corded temporal artery, and relief of the pain, which in some cases does not return, but in others recurs when the physiological effects of the drug have passed off. As individuals are affected differently by nitro-glycerine, I always begin with one minim of the one per cent. solution, but sometimes find it necessary to increase the dose to three or four minims to produce the desired effect. In several cases of asthma it has relieved the breathing in a most remarkable manner; the cases in which it answers are such as would be relieved by amyl-nitrite, but its effects are more marked and the relief is more durable.

One case of severe asthma, occurring in a patient suffering from chronic renal mischief and mitral deficiency, is worth special mentioning. I prescribed the one per cent. solution in the form of a minim to a drachm of water, and ordered two drachms to be taken every quarter of an hour till relief was obtained. My patient, however, had two large tablespoonfuls of the medicine given, instead of two teaspoonfuls. He said that the effect was wonderful; he thought his head was going to burst, but his breathing was effectually and permanently relieved, and that instantly. In this case, amyl-nitrite, although inhaled in large doses on previous occasions, had given very little relief. Since that time, several months ago, he has been threatened over and over again with his old attacks, but a dose of the medicine always staves it off.

In angina pectoris the relief given by nitro-glycerine is most complete; but as several cases have been reported in the journals, I need only mention it. The relief in these cases is not simply temporary ease from pain, but if the remedy be given thrice daily in gradually increasing doses, beginning with one minim of the one-per-cent. solution and steadily advancing to eight minims, the attacks lessen both in frequency and intensity. One of my patients, who has suffered severely from angina, always carries a bottle of the medicine in his pocket, and he tells me that by taking a dose of five drops when he is threatened with an attack it is always prevented.

I am trying it in some cases of epilepsy; but as yet my observations are not sufficiently advanced to be worth relating. I cannot see why, if it relieve the vaso-motor spasms in other diseases, it should not also have the same tendency in this most distressing disease; and since its regular use in angina seems to be curative, I have hopes that here we may have a similar effect. Again, if the "aura" gave sufficient warning, it might be worth while to try if a good dose would prevent an attack.

I have not had a chance of trying it in sea-sickness, but should think it might do good; and it would certainly have this advantage over amyl-nitrite, that all the other occupants of the cabin would not be compelled to inhale its fumes for some time afterward, which is the case if amyl-nitrite be used in the ordinary way, much to the annoyance of those who are well.—*Medical and Surg. Rep.*, May 8.

